# Management of non-urgent paediatric emergency department attendances by GPs: a retrospective observational study

**DOI:** 10.3399/bjgp20X713885

**Published:** 2020-12-01

**Authors:** Simon Leigh, Bimal Mehta, Lillian Dummer, Harriet Aird, Sinead McSorley, Venessa Oseyenum, Anna Cumbers, Mary Ryan, Karl Edwardson, Phil Johnston, Jude Robinson, Frans Coenen, David Taylor-Robinson, Louis W Niessen, Enitan D Carrol

**Affiliations:** Institute of Infection and Global Health;; Emergency Department;; School of Medicine, University of Liverpool, Liverpool.; School of Medicine, University of Liverpool, Liverpool.; School of Medicine, University of Liverpool, Liverpool.; School of Medicine, University of Liverpool, Liverpool.; Primary Care 24, Liverpool.; Primary Care 24, Liverpool.; Information Department, Alder Hey Children’s NHS Foundation Trust, Liverpool.; Information Department, Alder Hey Children’s NHS Foundation Trust, Liverpool.; School of Social and Political Sciences, University of Glasgow, Glasgow.; Department of Computer Science;; Department of Public Health and Policy;; Liverpool School of Tropical Medicine, Liverpool.; Institute of Infection and Global Health;

**Keywords:** antibiotics, cost-effectiveness, emergency care, paediatrics, primary care

## Abstract

**Background:**

Non-urgent emergency department (ED) attendances are common among children. Primary care management may not only be more clinically appropriate, but may also improve patient experience and be more cost-effective.

**Aim:**

To determine the impact on admissions, waiting times, antibiotic prescribing, and treatment costs of integrating a GP into a paediatric ED.

**Design and setting:**

Retrospective cohort study explored non-urgent ED presentations in a paediatric ED in north-west England.

**Method:**

From 1 October 2015 to 30 September 2017, a GP was situated in the ED from 2.00 pm until 10.00 pm, 7 days a week. All children triaged as ‘green’ using the Manchester Triage System (non-urgent) were considered to be ‘GP appropriate’. In cases of GP non-availability, children considered non-urgent were managed by ED staff. Clinical and operational outcomes, as well as the healthcare costs of children managed by GPs and ED staff across the same timeframe over a 2-year period were compared.

**Results:**

Of 115 000 children attending the ED over the study period, a complete set of data were available for 13 099 categorised as ‘GP appropriate’; of these, 8404 (64.2%) were managed by GPs and 4695 (35.8%) by ED staff. Median duration of ED stay was 39 min (interquartile range [IQR] 16–108 min) in the GP group and 165 min (IQR 104–222 min) in the ED group (*P*<0.001). Children in the GP group were less likely to be admitted as inpatients (odds ratio [OR] 0.16; 95% confidence interval [CI] = 0.13 to 0.20) and less likely to wait >4 hours before being admitted or discharged (OR 0.11; 95% CI = 0.08 to 0.13), but were more likely to receive antibiotics (OR 1.42; 95% CI = 1.27 to 1.58). Treatment costs were 18.4% lower in the group managed by the GP (*P*<0.0001).

**Conclusion:**

Given the rising demand for children’s emergency services, GP in ED care models may improve the management of non-urgent ED presentations. However, further research that incorporates causative study designs is required.

## INTRODUCTION

The total number of visits to emergency departments (EDs) in England exceeded 24 million in 2018^1^ and has risen by 42% since 2008,^2^ with over two-thirds of attendances taking place without GP referral or transfer by ambulance.^3^ Although these attendances may result from an acute medical problem, they may not always require immediate, specialised emergency medical care, with 20%–40% of ED visits having been classified as non-urgent.^4^^,^^5^ Increased concern regarding the potential severity of conditions,^6^ parental anxiety,^7^ and a perceived need for urgent treatment^7^^–^^10^ exacerbate this problem in children’s emergency medicine. Confidence in the quality and investigative ability of ED care,^7^ as well as difficulty obtaining primary care appointments,^11^ also plays a role; as such, it is estimated that one in two attendances for acute paediatric care could feasibly be managed in the community.^12^

A major challenge for staff in paediatric emergency care is to recognise children who are seriously ill, and the increasing use of EDs for non-urgent conditions makes this difficult; ED overcrowding is a major patient safety concern,^13^^,^^14^ which can result in suboptimal patient outcomes and even death.^15^^,^^16^

Given that an increasing number of non-urgent ED attendances are amenable to treatment in primary care, one of the key recommendations of a joint report published by the College of Emergency Medicine, Royal College of Paediatrics and Child Health, Royal College of Physicians, and Royal College of Surgeons is to co-locate primary care services within ED settings.^17^ Although the benefits of introducing GPs into EDs for managing non-urgent cases are well documented and include increased patient satisfaction,^18^^–^^20^ reduced waiting times,^19^ and reductions in invasive examinations,^21^ it is unclear whether this represents an efficient use of NHS resources, with the only economic analysis to date taking place in 1996.^22^ Building on the authors’ previous findings from a 6-month pilot scheme of the initiative,^19^ which assessed clinical and process outcomes, this retrospective observational study, conducted in one of Europe’s largest and busiest specialist paediatric EDs, assesses the impact of a primary care service located in an ED on waiting times, admissions, antibiotic prescribing rates, and healthcare costs. The aim was to determine the cost-effectiveness of the ED co-location of GP services.

**Table:** How this fits in

Many emergency department (ED) attendances are non-urgent, putting pressure on services and increasing caseloads. Having a GP available in the ED to manage non-urgent cases has previously been shown to improve efficiency and patient satisfaction, but it is unclear whether this demonstrates value for money. This large, non-randomised, observational study shows that children seen by the GP in the ED waited less time to be seen, had fewer inpatient admissions, and incurred lower healthcare costs, but experienced higher antibiotic prescribing than those managed by ED teams. As the demand for children’s emergency services is increasing, having a GP present in the ED may have a positive effect on how non-urgent paediatric cases are managed. Further research is, however, required.

## METHOD

### Study setting, population, and design

The study was conducted retrospectively in the ED of a large paediatric hospital located in the north-west of England. From 1 October 2015 until 30 September 2017, a GP employed by a Liverpool-based social enterprise delivering NHS services (Primary Care 24, formerly Urgent Care 24) was available in the ED as a separate but co-located service. The service ran from 2.00 pm until 10.00 pm, 7 days a week.

All children were initially evaluated by a qualified ED nurse using the Manchester Triage System (MTS).^23^ Low-acuity children triaged as non-urgent (categorised as MTS green without comorbidities) were labelled ‘GP appropriate’ and allocated to be seen by the GP during the operational hours of 2.00 pm–10.00 pm. Parents were not given a choice of allocation to the GP or otherwise but were informed of the decision, at which point they could refuse the service. Children referred to the ED by their own GP or a walk-in centre were ineligible for allocation to the GP in the ED service.

**Figure 1.**
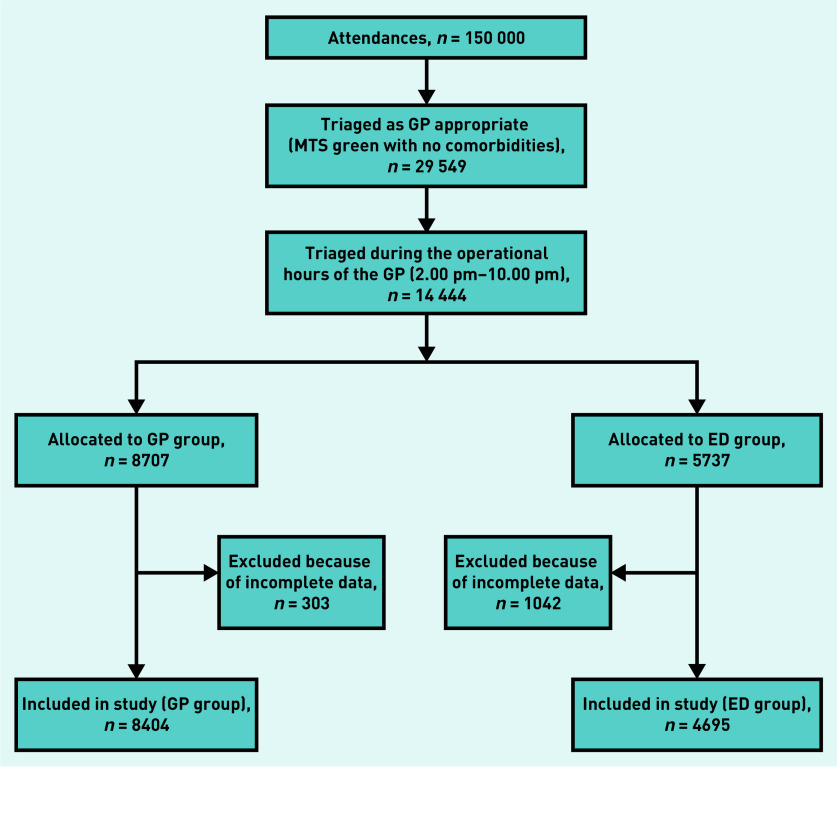
**Study recruitment process.** **ED = emergency department. MTS = Manchester Triage System.**

In instances of GP non-availability — namely, GP sickness — children triaged as ‘GP appropriate’, who would otherwise have been managed by onsite GPs, were instead managed by ED clinical staff, following the standard procedures of the service (the comparator group). This intervention presented an opportunity to evaluate a natural experiment, comparing both outcomes — antimicrobial prescribing, wait times (and achievement of the Department of Health and Social Care’s 4-hour target), and admission rates — and costs of children presenting to the paediatric ED with the same clinical urgency (MTS green) over the same time period (2.00 pm–10.00 pm, 7 days a week), differing only in terms of whether treatment was provided by ED teams or the co-located GP service. The study recruitment process is outlined in Figure 1.

Due to the retrospective observational nature of the study, in addition to primary outcome data, data concerning potential confounders were collected for all patients from both ED and GP service databases. For all cases, the following information were available:

- arrival and discharge date and time;
- final diagnosis;
- discharge status;
- antimicrobial prescribing; and
- attending physician.

Demographic data (age, sex, home postcode, and Index of Multiple Deprivation 2015 score) and clinical data (oxygen saturation, temperature, and pulse) were also collected. For those patients presenting with fever who received antibiotics, an assessment of whether antibiotic prescribing was clinically necessary was made, based on the retrospective application of an adapted algorithm from Herberg *et al*,^24^ details of which are provided in Supplementary Box S1.

### Statistical analysis

Patients triaged as ‘GP appropriate’ and managed by the GP service (exposed group) were compared with patients triaged as ‘GP appropriate’ and managed by ED staff over the same time period (control group), using an intention-to-treat approach. Descriptive statistics were generated for both groups. Differences in proportions were analysed using the χ^2^ test, with differences in continuous outcomes assessed via the Mann–Whitney *U* test. Multivariate logistic regression was used to estimate odds ratios (ORs) for binary outcomes, adjusted for baseline covariates that may have impacted outcomes, including whether children were re-attending the ED within a 5-day period or whether they had previously sought care from their community GP. Subgroup analyses were performed to account for covariates previously shown to impact the outcomes under consideration, including patient age,^25^ working diagnosis,^25^ and deprivation.^26^ All statistical analyses were conducted using Stata (version 12), with statistical significance defined at the 5% level.

### Costing and resource-use analysis

Healthcare resource use was calculated using a time-driven, activity-based costing (TDABC) approach, as used in previous health economic analyses conducted in the ED.^25^ TDABC identifies all instances and durations of interaction with health service personnel during a treatment episode and assigns time-dependent costs to each (triage, consultation, cannulation, and so on), based on stopwatch timing combined with the hourly salaries of the staff involved. Timing estimates and unit costs that were used for the patient-level healthcare costing are provided in Supplementary Tables S1 and S2. Finally, adding unit costs of consumables (including medicines) and tariff-based items (including investigations, radiography, and inpatient admission spells) provides an estimation of total resource use during a treatment episode. Further details of the methodology for the costing exercise are provided elsewhere.^25^ Societal costs to parents of waiting in the ED were also estimated by cross-referencing each responder’s postcode with hourly income data matched per lower layer super output area, which was obtained from the Office for National Statistics.^27^

All unit costs were in 2019 prices, with non-parametric bootstrapping (percentile method) used to generate 95% confidence intervals (CIs). Discounting of costs and outcomes was not required because of the short analysis timeframe. Probabilistic sensitivity analysis was also performed to test for robustness of conclusions regarding the impact of GP-led care on healthcare costs and outcomes. The distributions employed to explore parametric uncertainty are provided in Supplementary Table S3.

## RESULTS

### Baseline characteristics and recruitment

Between 1 October 2015 and 30 September 2017, 115 000 children visited the ED, of whom 14 444 were triaged as ‘GP appropriate’ (MTS green) between 2.00 pm and 10.00 pm, when the onsite GPs were in operation. Of these children, 1345 had incomplete or missing data, resulting in a cohort comprising 13 099 children. Table 1 shows the personal characteristics of those who were treated by a GP and those treated by ED staff; no statistically significant differences were observed in any of the demographic or clinical baseline characteristics.

**Table 1.** Characteristics of patients triaged as ‘GP appropriate’, attending the emergency department

**Variable**	**GP group, *N* = 8404**	**ED group, *N* = 4695**	**Total, *N* = 13 099**	***P*-value**
**Sex, *n* (%)**				0.206^a^
Male	4268 (50.8)	2541 (54.1)	6809 (52.0)	
Female	4136 (49.2)	2154 (45.9)	6290 (48.0)	

**Age category, *n* (%)**				0.785^a^
<3 months	613 (7.3)	319 (6.8)	932 (7.1)	
3–6 months	538 (6.4)	291 (6.2)	829 (6.3)	
7–12 months	1277 (15.2)	714 (15.2)	1991 (15.2)	
>1–3 years	3177 (37.8)	1779 (37.9)	4956 (37.8)	
4–10 years	2017 (24.0)	1174 (25.0)	3191 (24.4)	
≥11 years	782 (9.3)	418 (8.9)	1200 (9.2)	

**Age, years, median (IQR)**	2.2 (0.90–5.50)	2.15 (0.87–5.50)	2.17 (0.88–5.50)	0.624^b^

**Deprivation quintiles, *n* (%)^c^**				0.656^a^
1 (least deprived)	208 (2.5)	106 (2.3)	314 (2.4)	
2	456 (5.4)	253 (5.4)	709 (5.4)	
3	833 (9.9)	504 (10.7)	1337 (10.2)	
4	898 (10.7)	528 (11.2)	1426 (10.9)	
5 (most deprived)	5378 (64.0)	3058 (65.1)	8436 (64.4)	

**Diagnosis, *n* (%)**				n/a
Respiratory conditions	2070 (24.6)	1076 (22.9)	3146 (24.0)	
Gastrointestinal conditions	1410 (16.8)	695 (14.8)	2105 (16.1)	
Infectious disease	1194 (14.2)	695 (14.8)	1889 (14.4)	
Diagnosis not classifiable	530 (6.3)	946 (20.1)	1476 (11.3)	
ENT conditions	679 (8.1)	227 (4.8)	906 (6.9)	
Local infection	561 (6.7)	305 (6.5)	866 (6.6)	
Dermatological conditions	302 (3.6)	99 (2.1)	401 (3.1)	
Urological conditions (including cystitis)	256 (3.0)	128 (2.7)	384 (2.9)	
Allergy (including anaphylaxis)	263 (3.1)	100 (2.1)	363 (2.8)	
Head injury	190 (2.3)	45 (1.0)	235 (1.8)	
Fever	1289 (15.3)	643 (13.7)	1932 (14.7)	

**Pulse, beats/minute, median (IQR)**	127 (109–143)	125 (109–140)	126 (109–142)	0.864^b^

**Temperature, ºC, median (IQR)**	37 (36.6–37.6)	37 (36.6–37.6)	37 (36.6–37.6)	0.767^b^

**Oxygen saturation, %, median (IQR)**	99 (97–100)	99 (97–100)	99 (97–100)	0.558^b^

**Attended ED in last 5 days, *n* (%)**				0.14^a^
Yes	160 (1.9)	103 (2.2)	263 (2.0)	
No	8244 (98.1)	4592 (97.8)	12 836 (98.0)	

**Attended ED on a weekday, *n* (%)**				0.84^a^
Yes	5824 (69.3)	3301 (70.3)	9125 (69.7)	
No	2580 (30.7)	1394 (29.7)	3974 (30.3)	

**Attended ED during holiday period, *n* (%)^d^**				0.134^a^
Yes	2958 (35.2)	1592 (33.9)	4550 (34.7)	
No	5446 (64.8)	3103 (66.1)	8549 (65.3)	

a χ^2^.

b *Mann–Whitney* U *test.*

c *Deprivation data were based on postcodes. Many of the children attending the ED either had no postcode on file, incomplete postcodes, or were classed as Travellers, with postcodes that did not link to the Office for National Statistics database.* N*-values for GP group, ED group, and Total are 7773, 4449, and 12 222, respectively.*

d Holidays were in line with the English academic year and included half terms, Easter, Christmas, and winter holidays. ED = emergency department. ENT = ear, nose, and throat. IQR = interquartile range.

### Antibiotic prescribing

Rates of antibiotic prescribing were 15.1% in the GP group and 10.8% in the ED group (OR 1.42; 95% CI = 1.27 to 1.58; *P*<0.001) (see Supplementary Figure S1). Compared with children managed by ED teams, those managed by the GP who were seen and discharged within 1 hour had an OR of 3.32 (95% CI = 2.20 to 5.00) for being prescribed antibiotics. Children managed by the GP group who had fever at presentation experienced a 10.4% increase in antibiotic prescribing (27.1% versus 16.7%) (data not shown). Approximately 89.9% of children with fever receiving antibiotics in the GP group, compared with 75.9% in the ED group, displayed no evidence of bacterial foci (see Supplementary Table S4).

### Wait times

The median duration of stay in the ED was 39 min (interquartile range [IQR] 16–108 min) for the GP group, compared with 165 min (IQR 104–222 min) for the ED group (*P*<0.005) (data not shown). Management by the onsite GP was associated with statistically significantly reduced odds of breaching the UK Department of Health and Social Care’s 4-hour waiting standard (OR 0.10; 95% CI = 0.08 to 0.13; *P*<0.001); 98.6% of children in the GP group and 88.4% of those in the ED group were discharged or admitted within 4 hours (data not shown).

### Admission to hospital and discharge status

The odds of being admitted were statistically significantly lower (84.0%) for children managed by the GP (OR 0.16; 95% CI = 0.13 to 0.20; *P*<0.001) than those managed by ED staff (data not shown). Short-stay admissions of <6 hours were reduced by 84.7%, 6–24-hour admissions by 86.5%, and admissions exceeding 24 hours by 78.7% for those seen by the GP, when compared with the group managed by ED staff. Children in all age groups and all diagnostic groups were statistically significantly more likely to be admitted to hospital if managed by ED clinical teams (all *P*<0.001) (data not shown). The grade of the ED clinician managing the child had no impact on admission rates (data not shown).

In total, 95.9% of children in the GP group were discharged with no further action or advised to seek follow-up with their own GP, compared with 76.0% in the ED group (Table 2). Outpatient referrals were equivalent across groups, with 107 (1.3%) children in the GP group and 103 (2.2%) children in the ED group referred, but 9.7% of children in the ED group left the ED before being seen, compared with 1.2% in the GP group (Table 2).

**Table 2.** Discharge status of children by treatment group

**Discharge status**	**GP group,^a^*n* (%)**	**ED group,^b^*n* (%)**	**Total,^c^*n* (%)**
Own GP follow-up	2312 (27.5)	287 (6.1)	2599 (19.8)
Discharged with no further action	5745 (68.4)	3282 (69.9)	9027 (68.9)
Admitted	117 (1.4)	374 (8.0)	491 (3.7)
Outpatient	107 (1.3)	103 (2.2)	210 (1.6)
ED clinic	3 (<0.1)	59 (1.3)	62 (0.5)
Community follow-up	1 (<0.1)	0 (0.0)	1 (<0.1)
Left before seen	100 (1.2)	455 (9.7)	555 (4.2)
Left following advice	1 (<0.1)	5 (0.1)	6 (<0.1)
Left refusing treatment	6 (0.1)	117 (2.5)	123 (0.9)
Other	5 (0.1)	13 (0.3)	18 (0.1)
N/A	7 (0.1)	0 (0.0)	7 (0.1)

a N *= 8404.*

b N = 4695.

d N = 13 099. ED = emergency department.

### Healthcare and societal costs of ED management

The mean cost of treatment episodes for the GP group was 115.24 GBP (95% CI = 20.50 to 351.67 GBP), compared with 141.16 GBP (95% CI = 11.78 to 539.94 GBP) among those managed by ED clinicians (*P*<0.001) (data not shown). Both groups recorded similar costs attributable to medications prescribing, and investigations (Table 3). Costs associated with staff salaries (receptionist, nurse, and doctor) were much higher in the GP group than in the ED group, but inpatient admission costs were statistically significantly lower (*P*<0.001) (Table 3); this owed primarily to a 75.3% reduction in median inpatient duration (0.22 days versus 0.89 days) (data not shown). Societal costs were increased by 27.18 (46.87 versus 19.69 GBP) in the ED group, compared with the GP group (Table 3).

**Table 3.** Breakdown of cost types per patient in the GP and ED treatment groups

**Cost type**	**Costs, GBP**			***P*-value^a^**
	**GP group**	**ED group**	**Difference**	
Staff salaries	82.81	46.00	36.81	0.001
Observation/inpatient	28.86	89.28	60.42	0.001
Prescribed medications	3.09	3.29	0.20	0.385
Investigations	0.43	2.77	2.34	0.001
Societal^b^	19.69	46.87	27.18	0.001

a *Mann–Whitney* U *test.*

b Calculated as a function of total time in the ED, expressed in terms of forgone wages and productivity by parents and carers. ED = emergency department. GBP = Great British pound.

### Subgroup analyses

Subgroup analyses for all outcomes are provided in Table 4 and Supplementary Box S2.

**Table 4.** Comparative costs per patient and outcomes by subgroup

**Variable**	**Costs, GBP**				**Antibiotics, %**				**4-hour target, %**				**Inpatient, %**			

	**GP group**	**ED group**	**All**	***P*-value**	**GP group**	**ED group**	**All**	***P*-value**	**GP group**	**ED group**	**All**	***P*-value**	**GP group**	**ED group**	**All**	***P*-value**
**Working diagnosis**																
Fever, *n* = 1926	93.78	69.76	86.69	<0.001	27.1	16.7	23.5	<0.001	98.5	87.5	94.6	<0.001	1.1	4.5	2.3	<0.001
Infectious disease, *n* = 1889	92.18	123.29	103.94	<0.001	5.7	5.9	5.7	0.578	98.7	89.1	94.7	<0.001	0.7	9.9	4.4	<0.001
Gastrointestinal, *n* = 2105	89.49	120.77	104.76	<0.001	0.5	0.6	0.6	0.891	98.8	86.2	94.4	<0.001	1.0	8.6	3.9	<0.001
Respiratory *n* = 3146	87.52	89.40	88.16	0.897	16.2	10.2	14.3	<0.001	98.9	86.3	94.3	<0.001	0.5	6.5	2.7	<0.001
Local infection, *n* = 866	92.97	88.26	91.34	0.521	40.3	39.9	40.2	0.978	98.4	86.4	93.9	<0.001	0.7	4.1	2.0	<0.001
ENT, *n* = 906	86.78	111.90	92.30	<0.001	41.5	35.7	40.1	0.298	97.8	86.8	95.0	<0.001	0.0	2.8	0.7	<0.001

**Age**																
<3 months, *n* = 932	99.49	242.54	152.88	<0.001	5.2	5.6	5.4	0.947	99.2	87.9	95.2	<0.001	1.2	14.3	6.2	<0.001
3–6 months, *n* = 829	135.55	196.38	162.38	<0.001	8.8	8.2	8.6	0.935	98.5	90.1	95.2	<0.001	2.3	7.1	4.5	<0.001
6–12 months, *n* = 1991	101.04	95.29	100.60	<0.001	13.1	8.6	11.5	0.012	98.4	89.5	94.4	<0.001	1.6	7.8	4.2	<0.001
1–3 years, *n* = 4956	99.83	116.47	109.70	<0.001	18.2	11.5	15.7	<0.001	98.6	87.6	94.2	<0.001	1.1	7.1	3.6	<0.001
4–10 years, *n* = 3191	118.36	130.14	132.08	<0.001	16.8	13.4	15.5	0.037	98.6	89.5	94.6	<0.001	1.4	5.7	3.3	<0.001
≥1 years, *n* = 1200	115.39	238.72	157.93	<0.001	13.9	10.4	12.9	0.07	98.5	86.0	93.8	<0.001	1.6	7.7	3.9	<0.001

**Deprivation quintile**																
1 (most deprived), *n* = 8436	111.56	150.61	126.23	<0.001	15.4	10.3	13.5	<0.001	98.6	87.3	94.3	0.005	1.4	7.8	3.8	<0.001
2, *n* = 1426	108.43	150.48	124.33	<0.001	16.6	11.5	14.7	0.009	99.4	88.6	95.4	<0.001	1.2	8.9	4.2	0.003
3, *n* = 1337	94.17	170.70	124.10	<0.001	14.8	11.0	13.3	0.047	98.3	88.8	94.6	<0.001	1.7	7.7	4.0	<0.001
4, *n* = 709	104.17	92.69	99.98	<0.001	12.7	12.9	12.8	0.921	98.2	88.9	94.8	<0.001	1.5	5.7	3.1	<0.001
5 (least deprived), *n* = 314	115.55	189.99	141.29	<0.001	14.9	17.3	15.7	0.582	97.6	89.1	94.7	<0.001	1.4	10.9	4.7	<0.001

a *Significance determined via Mann–Whitney* U *test. ED = emergency department. ENT = ear, nose, and throat. GBP = Great British pound.*

### Sensitivity analysis

**Figure 2.**
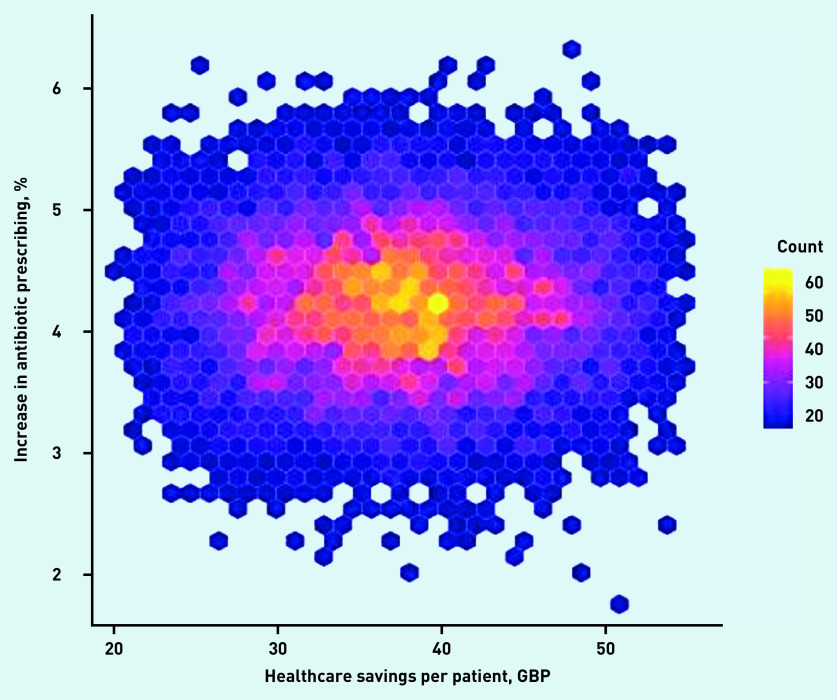
**Variability in health service savings and antibiotic use following introduction of GP to emergency department.** ***Count = number of Monte Carlo simulations in which the cross-section of the*** **x *****- and*** **y *****-axis occurred. GBP = Great British Pound.***

Probabilistic sensitivity analysis utilising the distributions provided in Supplementary Table S3 suggested an 86.0% probability that GP-led care would result in a saving of at least 30 GBP per patient. Similarly, there was a 98.3% probability that treatment by GPs in the ED would increase antibiotic prescribing by at least 3% (Figure 2).

## DISCUSSION

### Summary

During a 2-year natural experiment, in which a GP service was co-located in a busy paediatric ED for non-urgent admissions, patients being managed by GPs instead of ED staff resulted in lower treatment costs, fewer hospital admissions, and fewer patients exceeding the 4-hour waiting target; however, those seen by the GP were subject to higher rates of antimicrobial prescribing.

### Strengths and limitations

To the best of the authors’ knowledge, the study presented here, conducted among a large and representative ED cohort over a 2-year period, is the first to assess the combined clinical, process-based, and economic impact of introducing a GP service to a paediatric ED in the UK. The authors have made use of a natural experiment and routinely collected data to pragmatically evaluate the impact of GP co-location in one of Europe’s largest and busiest specialist paediatric EDs. Although this was a retrospective observational study, the treatment groups were almost identical in terms of demographics and case mix, which have been previously shown to affect the outcomes under consideration.^25^ This limited the likelihood of confounding bias, thereby providing generalisable insights regarding the management of non-urgent presentations to EDs. Furthermore, although observational, the approach taken to estimate costs was highly thorough and representative of real-world management, including details such as nursing time required to prepare and provide medications, and clinical time required to order and interpret investigations.

The study presented here does have some limitations. The authors did not collect data on several factors that may have affected both ED and GP staff workload, including: how busy the department was at any given time; the number of staff on shift; and the availability and capacity of connected departments, such as pathology and radiology, which may have affected the ability for GPs and ED clinicians to treat and investigate the children included efficiently. In addition, although every effort was made to eliminate sources of bias, including the large patient numbers and subsequently balanced baseline characteristics, the retrospective nature of the study and lack of randomisation does leave the opportunity for unknown causes of bias that could not be adjusted for.

Higher rates of incomplete data capture and exclusion for the ED group very likely did not impact the findings. These seemed to be missing at random in verification samples; however, the authors can neither confirm this with certainty, nor determine how these patients would have affected the detailed findings of the study.

Finally, the fact that the operational hours of the GP service only covered a third of the operating hours of the ED (2.00 pm-10.00 pm) means that generalisability of the findings could be limited as it cannot be guaranteed that similar patterns of care would be observed overnight when services, diagnostics, and access to radiography are limited.

### Comparison with existing literature

Prior interventional analyses and systematic reviews have suggested that the co-location of GPs in EDs may not have a significant impact on reducing the cost of care per patient^28^^,^^29^ but may, in fact, increase costs because of extra personnel.^29^ However, the findings presented here — in the largest cohort to date of which the authors are aware — suggest otherwise. Despite personnel costs increasing, children requiring non-urgent health care managed by GPs experienced significant reductions in total costs of management, predominantly resulting from reductions in inpatient admission, investigations, and radiography; this has also been observed in similar studies.^21^^,^^22^^,^^30^ This difference was most pronounced among younger children (aged <6 months), for whom healthcare costs were reduced by almost 60% and in whom, understandably, ED staff are known to be most cautious.^25^

In EDs that are frequently overcrowded, the significant reduction in activities associated with waiting (observation, investigations, and radiography) as observed in the GP group, may have a significant effect on patient flow through the ED, resulting in reductions in waiting times and increases in patient satisfaction. This could have major implications for NHS trusts, as breaching the target of resolving at least 95% of the attendances within 4 hours can have serious negative economic consequences for hospitals.^31^ The increase in achievement of the 4-hour standard from 88.4% in the ED group to 98.6% in the GP group, therefore, also has the potential to save NHS trusts money in the short-to-medium term — possible savings that were not captured in this analysis. However, a potential limitation, observed in both this study and the authors’ previously published pilot study,^19^ is that a substantial number of patients managed by GPs were subsequently referred to their own GP for further follow-up; this may simply shift some of the burden to primary care. As such, the impact on the whole system of GP in the ED models of care still requires further investigation.

Finally, although GP-led care for non-urgent attendances resulted in several statistically significant benefits, the resulting increase in antibiotic prescription was also statistically significant. There are considerable clinical policy pressures on GPs not to miss sepsis, meningitis, or other illnesses that are serious but rare, often a result of diagnostic uncertainty,^25^ which may push practitioners to prescribe as a precaution.^25^^,^^32^^,^^33^ A previous study found that 44% of GPs might prescribe antibiotics to terminate a consultation;^34^ implicit in this finding is the potential effect of the increasingly tight time constraints under which GPs work, and the number of children seen over relatively short periods of time. Findings in relation to patients seen by a GP receiving higher rates of microbial prescribing corroborate those of the authors’ previous and much smaller study, which did not include a health economic analysis.^19^ In the study presented here, children managed by the GP who were seen and discharged within 1 hour were three times more likely to be prescribed antibiotics, compared with children seen and discharged within a similar period who were managed by ED clinicians. Consultation time and GP workload have been shown to be associated with higher antibiotic prescription rates^35^ and it is worth noting that, in this study, the GP managed almost twice as many non-urgent cases as ED clinicians over the same period. In Norway, a study found that GPs who saw more patients per year prescribed more antibiotics than those with fewer patients;^36^ this was echoed in a qualitative study of GPs and nurse prescribers in the UK.^35^

Advances in diagnostic technologies, such as rapid point-of-care (POC) testing, may play a role in reducing unnecessary antibiotic prescribing. POC C-reactive protein testing has been shown to reduce antibiotic prescribing in UK primary care clinics for patients with chronic obstructive pulmonary disease.^37^ Prior studies have also suggested community antibiotic stewardship by pharmacists,^38^ and prescribing or social norm feedback as part of continued GP education^35^^,^^39^ or primary care accreditation schemes,^40^ as means of reducing antimicrobial prescribing. Given the success of these initiatives in reducing antibiotic use in routine practice, coupled with low expected costs of implementation and GPs being easily accessible in a single hospital setting, there is every possibility to reduce antibiotic use.

### Implications for research and practice

Given the increasing demands on emergency care, integrative care approaches are a plausible means of increasing capacity and caseload management, particularly given the non-urgent nature of many attendees to the ED. The results of this large-scale natural experiment showed that children seen by a GP in the ED waited less time, had fewer inpatient admissions, and lower costs, but experienced higher antibiotic prescribing than those treated by ED teams. However, further research incorporating causative study designs are required to determine causality between GP management and these outcomes.
